# The changing landscape of palliative epilepsy surgery for Lennox Gastaut Syndrome

**DOI:** 10.3389/fneur.2024.1380423

**Published:** 2024-03-07

**Authors:** Ruba Al-Ramadhani, Jasmine L. Hect, Taylor J. Abel

**Affiliations:** ^1^Department of Pediatrics, Division of Child Neurology, University of Pittsburgh, Pittsburgh, PA, United States; ^2^Department of Neurological Surgery, University of Pittsburgh, Pittsburgh, PA, United States; ^3^Department of Bioengineering, University of Pittsburgh, Pittsburgh, PA, United States

**Keywords:** Lennox Gastaut Syndrome, corpus callosum ablation, vagus nerve stimulation, deep brain stimulation, responsive neurostimulation

## Abstract

Lennox Gastaut Syndrome (LGS) is characterized by drug-resistant epilepsy that typically leads to decreased quality of life and deleterious neurodevelopmental comorbidities from medically refractory seizures. In recent years there has been a dramatic increase in the development and availability of novel treatment strategies for Lennox Gastaut Syndrome patient to improve seizure. Recent advances in neuromodulation and minimally invasive magnetic resonance guided laser interstitial thermal therapy (MRgLITT) have paved the way for new treatments strategies including deep brain stimulation (DBS), responsive neurostimulation (RNS), and MRgLITT corpus callosum ablation. These new strategies offer hope for children with drug-resistant generalized epilepsies, but important questions remain about the safety and effectiveness of these new approaches. In this review, we describe the opportunities presented by these new strategies and how each treatment strategy is currently being employed. Next, we will critically assess available evidence for these new approaches compared to traditional palliative epilepsy surgery approaches, such as vagus nerve stimulation (VNS) and open microsurgical corpus callosotomy (CC). Finally, we will describe future directions that would help define which of the available strategies should be employed and when.

## Introduction: the need for more effective, albeit less morbid approaches

Lennox Gastaut Syndrome (LGS) is a severe childhood epilepsy syndrome that is typically drug-resistant and diagnosed before the age of 18. The diagnosis of LGS is based on the presence of certain clinical criteria, including various seizure types, primarily tonic (stiffening), but also atonic (drop) and atypical absence (staring), seizures are typically frequent and difficult to control pharmaceutically (1). Conventional medical treatments often fail to control seizures, leading to the need for alternative strategies to improve seizure frequency and comorbidities such as cognitive and behavioral impairments (1, 2).

Observational studies and international guidelines support a role for palliative epilepsy surgery for children with LGS (3–5). However, despite the availability of different surgical options (e.g., VNS and CC) it remains unclear how and when to employ different available surgical strategies. Moreover, recent surgical treatments such as deep brain stimulation (DBS) or responsive neurostimulation (RNS) for neuromodulation of the centromedian thalamic nucleus (CM-DBS; CM-RNS), as well as CC ablation provide new palliative surgery options, however little evidence is available about their safety and effectiveness. The availability of numerous surgical options for epilepsy teams and LGS patients and their epilepsy treatment team presents unique decision-making challenges. For example, preliminary data suggests that neuromodulation via DBS or RNS may be more effective than VNS (6), yet patients and families are often hesitant to consider an intracranial neurosurgery operation over traditional VNS. Similarly, open microsurgical CC is a well-established method for reducing drop attack seizures, but there is now also the option of less invasive CC ablation which avoids the potential complications of craniotomy and decreases length of hospitalization for patients (7). However, this approach is still relatively novel and high-quality evidence demonstrating its noninferiority to CC is not yet available (8).

## Traditional approaches: vagus nerve stimulation and corpus callosotomy

Vagus nerve stimulation (VNS) and corpus callosotomy (CC) are palliative therapies used to reduce seizure burden and improve quality of life in Lennox Gastaut Syndrome (LGS) (9, 10). VNS has been approved in the US since 1997 and is effective in reducing seizures by more than 50% with longer device usage (11). VNS also has a positive effect on epilepsy psychiatric comorbidity, including improvements in depression and overall mood (12). Side effects and complications of VNS are rare, with an incidence of less than 5%, and are usually related to the surgical intervention (13).

In comparison, CC is a palliative surgery that primarily targets drop attacks (atonic or bilateral tonic seizures). Drop attacks can cause serious injuries and have a significant impact on quality of life due to their abrupt onset of semiology and lack of warning signs. By disconnecting the corpus callosum, CC can reduce symptom severity by disrupting rapid generalization of seizures to reduce seizure morbidity and improve quality of life. CC requires craniotomy which carries risks such as infection, hemorrhage, and infarction (14). Hemiparesis, akinesia, mutism, and aggression have all been reported as major transient complications following disconnection in both CC and CC ablation. Importantly, some of the traditionally reported complications (e.g., hemiparesis) may be due to retraction on motor, cingulate gyrus, and premotor cortex en route to the corpus callosum, rather than disconnection of the corpus callosum itself, and may be less likely with minimally invasive CC ablation that does not require retraction. CC effectiveness varies, with complete callosotomy typically providing better seizure control than partial callosotomy (15).

While there are no comparative effectiveness studies of VNS vs. CC ablation in children with atonic seizures. A recent meta-analysis comparing CC ablation to VNS demonstrates that CC has a notable advantage over VNS in treating atonic seizures in pediatric patients. This benefit, however, should be carefully measured against the increased risks of re-operation and disconnection syndrome (10). It is important to note, however, that often both surgeries are utilized in patients with severe LGS and that their effects are not mutually exclusive. Abel et al. performed a decision analysis suggesting that while CC is more effective, VNS may be more cost-effective at one-year follow-up (16). While CC may have better seizure outcomes for drop attacks, considering post-op complications, recovery time, and cost efficiency, VNS may be a favorable first-line treatment option for DRE in LGS patients, despite being associated with cessation of drop attacks in only 20% of patients.

## Novel minimally invasive approaches: laser interstitial thermal therapy corpus callosum ablation

**Figure 1:**
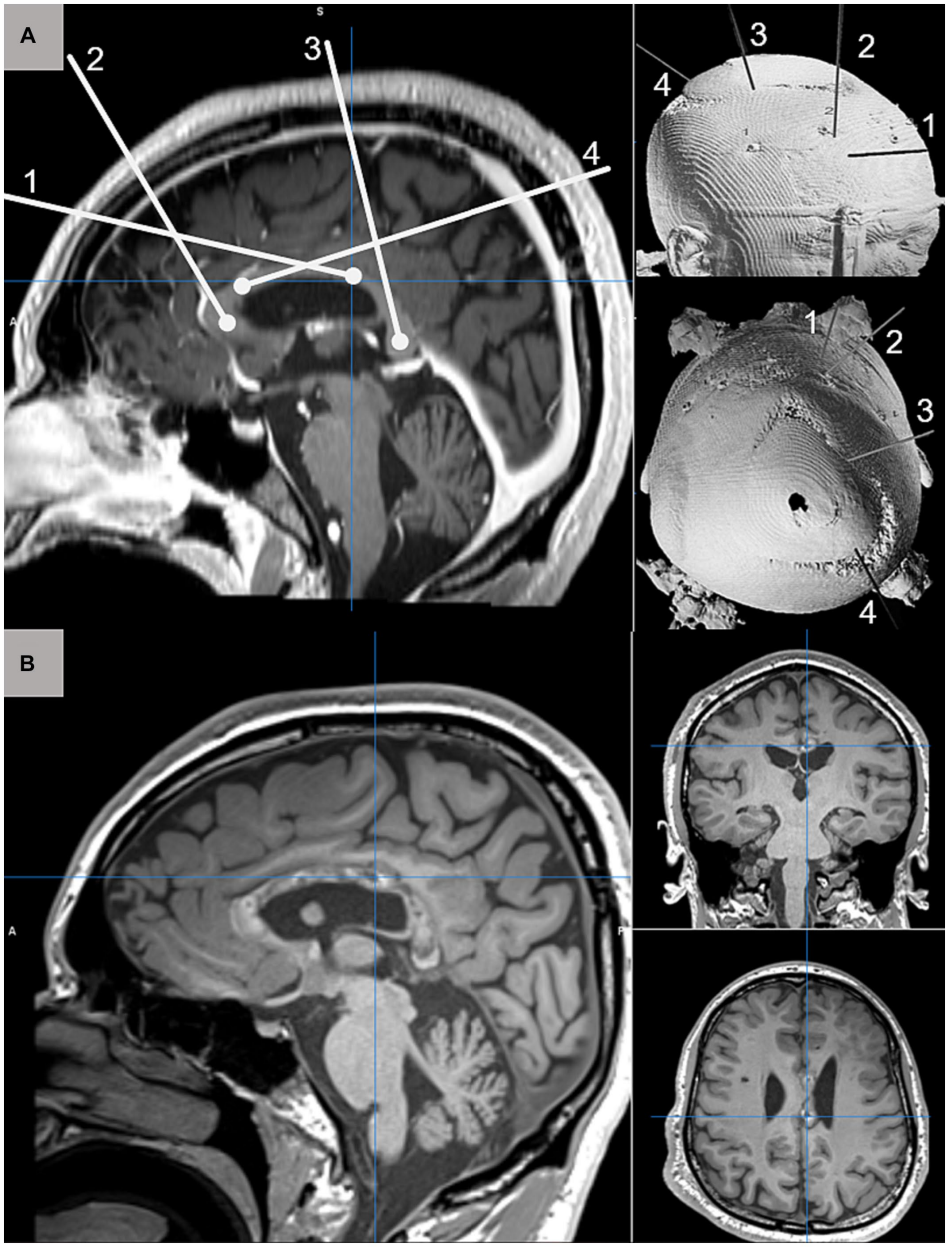
**(A)** Schematic depiction of four MRgLITT trajectories for complete CC ablation targeting the genu (1), posterior body (2), anterior body (4), and splenium (3) displayed on sagittal T1-weighted pre-operative MRI (left) and 3D CT reconstruction from anterolateral and superior perspectives. **(B)** Postoperative T1-weighted MRI acquired 3-months after complete CC ablation demonstrating normal postoperative changes along the extent of targeted callosal white matter.

Magnetic resonance guided laser interstitial thermal therapy (MRgLITT) is a minimally invasive alternative to traditional epilepsy surgery in which a laser fiber is inserted into the brain target using standard stereotactic technique to create a lesion that can be precisely monitored in real time with MR thermography to ensure the safety of surrounding brain structures (Figure 1). MRgLITT has been shown to be safe and effective for certain etiologies such as hypothalamic hamartoma (17–19).

Traditional corpus callosotomy (CC) is performed via open craniotomy and carries risks of complications, leading to prolonged post-operative recovery time. CC ablation has emerged as a potential alternative, with early case series and cohort studies suggesting similar efficacy to open CC but with fewer complications and shorter hospitalization (8, 20–22). Multiple centers have reported several case series about CC ablation safety and effectiveness (21, 23, 24) for reducing seizure frequency. Awad and Kaiser et al. reported 10 patients who underwent 11 MRI-guided CC ablation and concluded that it offers a minimally invasive alternative approach to CC with minor intraoperative complication and faster recovery time (25).

Although complete CC may have better seizure outcomes compared to partial CC, it also carries higher risks of post-operative complications, such as disconnection syndrome. Despite the advantages of minimally invasive approaches like CC ablation, existing evidence suggests the risk of callosal disconnection-related complications remains unchanged (26). This makes sense given that a complete disconnection of the CC is still occurring and much of the morbidity of CC is due to callosal disconnection. Additionally, CC primarily targets drop attacks and may not be sufficient to change the current clinical approach for DRE in LGS patients. However, the minimally invasive approach of CC ablation may be better tolerated in LGS patients with multiple comorbidities and may be considered more acceptable to patients and caregivers who prefer a minimally invasive approach.

## Neuromodulation: a new landscape with innovative approaches, yet limited evidence

**Figure 2:**
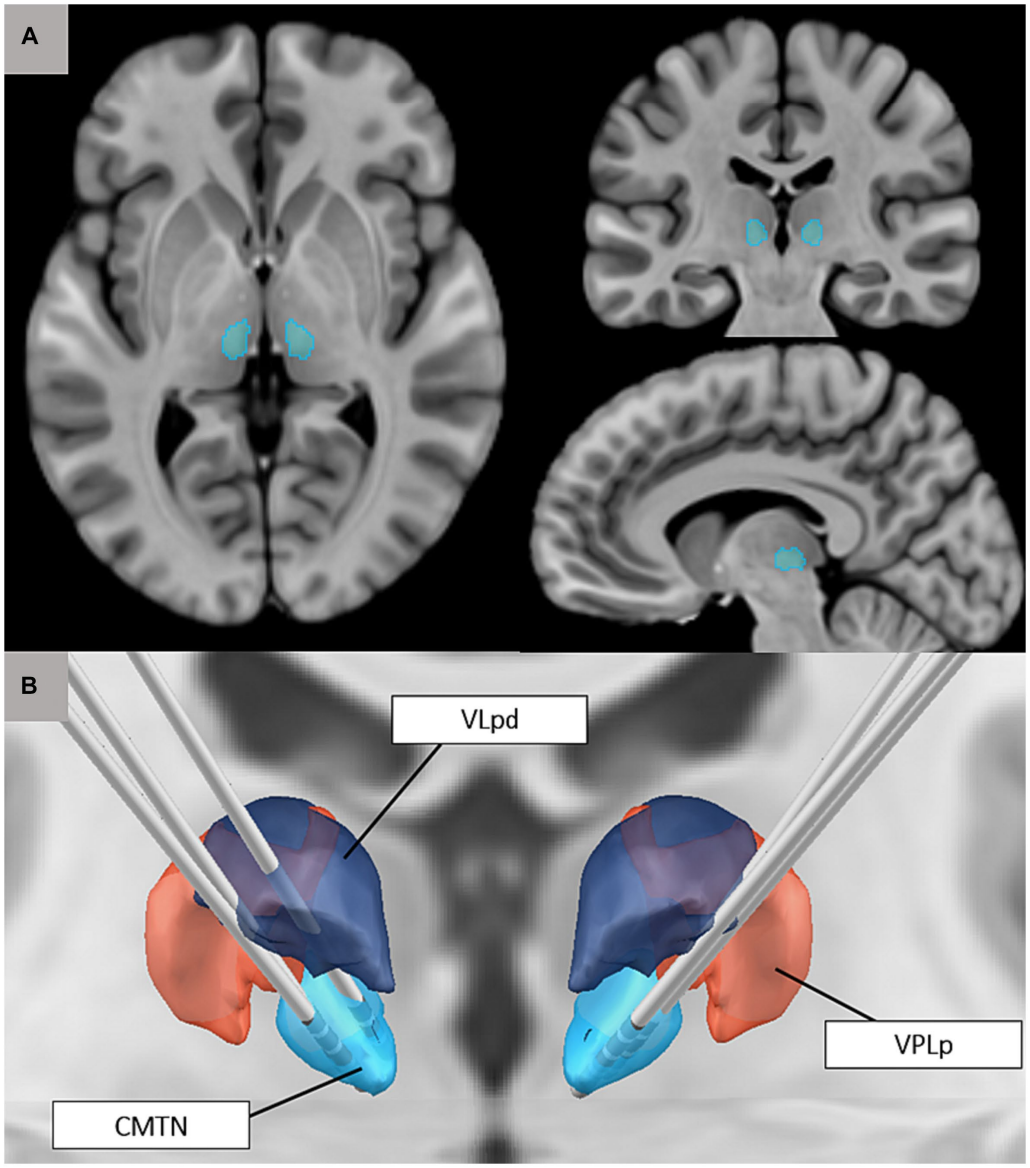
**(A)** Visualization of the centromedian thalamic nucleus based on the Morel thalamic atlas on adult anatomy (T1-weighted MNI template image). **(B)** 3D rendering of the CMTN (blue) in relationship to ventral posterolateral nucleus (VPLp; red) and posterior dorsal part of the ventral lateral nucleus (VLpd; purple). Also pictured are reconstructions of bilateral CM-RNS trajectories for three cases (27).

Centromedian thalamic nucleus (CMTN) stimulation, with CM-DBS or CM-RNS, is a newer surgical neuromodulation method used to help control seizures in patients with LGS involving implantation of electrodes in the bilateral CMTN (Figure 2). The device is powered and programmed by a pulse generator implanted in the chest wall. Although the mechanism by which neuromodulation of the CMTN terminates seizures is not entirely understood, it is hypothesized that by interfering with the low-frequency ictal thalamocortical recruitment, it may increase consciousness and stop seizure discharges from spreading (28).

The CMTN is characterized by widespread connections throughout the brain. Specifically, the lateral portion of the CM has connections with the premotor, motor, and primary somatosensory cortices. White matter tractography studies have also suggested that the CM is associated with the anterior insula and frontal operculum networks, and projects to other thalamic nuclei, including the reticular nucleus. This extensive network of connections makes the CM an attractive target for neuromodulation of seizure networks of generalized epilepsy (29). Increased firing in the thalamocortical pathway can lead to sustained firing patterns of thalamic nucleus reticularis cells, resulting in longer inhibitory potentials.

The ESTEL study, the only prospective, double-blind, randomized trial of CM-DBS in LGS patients, found a significant decrease in electrographic seizures and a reduction in absolute seizure counts after 3 months of stimulation (30). CMTN neuromodulation via CM-DBS is generally considered a safe and effective treatment option for LGS patients who have not responded well to other treatments. However, like any surgery, carries risks such as infection, hemorrhage, and brain tissue injury.

Similar to CM-DBS, CM-RNS is also a type of neuromodulation that involves implanting electrodes in the CMTN to modulate seizure networks. Unlike DBS, which sends pre-programmed stimulation to target areas, RNS allows for continuous monitoring of electrical activity in target areas and only deploys electrical pulses to the CMTN when seizures are detected. RNS is a relatively new neuromodulation treatment option for patients with DRE, and it has been used primarily for focal epilepsy, in which the seizure onset zone is identified within eloquent cortex and cannot be resected. While there is no formal trial evaluating CM-RNS for seizure control in LGS patients, it has been suggested that CM-RNS may reduce thalamic kindling-related adverse effects as seen with CM-DBS (28, 31). In a report by Kwon et al., two patients with LGS and DRE experienced significant reductions in seizure frequency and severity, as well as improvements in behavioral, cognitive, and quality of life outcomes with CM-RNS (31, 32).

Alcala-Zermino et al. investigate the use of CM-DBS with or without simultaneous anterior DBS (ANT-DBS) in 16 children and adults with difficult-to-treat generalized, multifocal, posterior origin, and diffuse onset DRE, five of whom had a diagnosis of LGS. Patients had a median monthly seizure rate of 323 (33). The treatment was found to be both safe and effective and demonstrated a median seizure frequency reduction of 58 and 63% of patients experienced at least a 50% reduction of seizure frequency, although outcomes did not differ for CM + ANT-DBS vs. CM-DBS. Quality of life and overall satisfaction improved in 56% of all patients. According to the study, simultaneous CM + ANT stimulation may desynchronize cortical and thalamocortical activity and reduce seizures from frontal and temporal structures connected to the Papez circuit. The significant reduction in seizure frequency typically led to notable improvements in neurodevelopmental outcomes for patients with LGS. These patients frequently have developmental delays and cognitive impairments, which can be exacerbated by frequent seizures. Research suggests patients’ cognitive abilities may even improve as a result of seizure reduction, such as increased attention span and alertness (5). These neurodevelopmental advances highlighted to the potential benefits associated with the newer therapeutic modalities for patients with LGS and other challenging epilepsies.

## A road forward: real-world studies are needed to determine best treatment algorithms

The availability of numerous surgical treatment options presents opportunities and challenges for epilepsy teams caring for children with LGS. Comparative effectiveness research can provide a way forward in evaluating and comparing the outcomes of different surgical treatments. This research can help guide clinical decision-making and improve patient outcomes by identifying the most effective and safe surgical strategies for LGS.

A major limitation of existing knowledge of the effectiveness of available treatments is that studies rely on seizure outcomes, but do not comprehensively assess quality of life. To that end, the significance of patient-reported outcomes (PROs) in determining the effectiveness of epilepsy surgery and neuromodulation outcomes is widely acknowledged. PROs provide clinicians with direct reports on a patient’s health status, allowing them to capture the broader effects of treatment beyond seizure frequency. Because epilepsy is a complex disorder that affects many aspects of patients’ lives, carefully designed questionnaires are required to quantify patient treatment experience and treatment impacts to understand the value of novel treatments. Various reviews on various aspects of PROs guide the selection of appropriate measures for clinical studies.

Neuromodulation therapy is growing as an alternative for refractory epilepsy patients. RCTs must be used to evaluate and compare the efficacy of different techniques, offer patients with evidence-based treatment alternatives, and guide clinical practice. RCTs can also detect potential side effects and provide data on safety and tolerability. However, RCTs are not always feasible or ethical, especially when the intervention involves significant risks, or the patient population is small. In these cases, comparative effectiveness research can be essential to compare relative efficacy of different surgical approaches, whether curative or palliative.

## Author contributions

RA-R: Writing – original draft, Writing – review & editing. JH: Writing – review & editing. TA: Supervision, Writing – review & editing.
